# Role of the Lymphotoxin/LIGHT System in the Development and Maintenance of Reticular Networks and Vasculature in Lymphoid Tissues

**DOI:** 10.3389/fimmu.2014.00047

**Published:** 2014-02-11

**Authors:** Theresa T. Lu, Jeffrey L. Browning

**Affiliations:** ^1^Autoimmunity and Inflammation Program and Pediatric Rheumatology, Hospital for Special Surgery, New York, NY, USA; ^2^Department of Microbiology and Immunology, Weill Cornell Medical College, New York, NY, USA; ^3^Department of Microbiology and Section of Rheumatology, Boston University School of Medicine, Boston, MA, USA

**Keywords:** lymphotoxin-beta receptor, reticular stroma, high endothelial venules, reactive lymph node, follicular dendritic cell, fibroblastic reticular cells

## Abstract

Lymphoid organs are meeting zones where lymphocytes come together and encounter antigens present in the blood and lymph or as delivered by cells migrating from the draining tissue bed. The exquisite efficiency of this process relies heavily on highly specialized anatomy to direct and position the various players. Gated entry and exit control access to these theaters and reticular networks and associated chemokines guide cells into the proper sections. Lymphoid tissues are remarkably plastic, being able to expand dramatically and then involute upon resolution of the danger. All of the reticular scaffolds and vascular and lymphatic components adapt accordingly. As such, the lymph node (LN) is a wonderful example of a physiologic remodeling process and is potentially a guide to study such elements in pathological settings such as fibrosis, chronic infection, and tumor metastasis. The lymphotoxin/LIGHT axis delivers critical differentiation signals that direct and hone differentiation of both reticular networks and the vasculature. Considerable progress has been made recently in understanding the mesenchymal differentiation pathways leading to these specialized networks and in the remodeling that occurs in reactive LNs. In this article, we will review some new advances in the area in terms of developmental, differentiation, and maintenance events mediated by this axis.

## Introduction

The functional specialization of the reticular scaffolds and the vascular and lymphatic vessels in lymphoid organs is essential for the exquisite sensitivity and efficiency of the immune system. These various stromal elements have been increasingly well studied over the last decade with major advances occurring in the description of their developmental lineages and differentiation as well as their activation and adaptation in reactive lymphoid organs. This field has been extensively reviewed (1–12). In general, the stromal cells in the secondary lymphoid organs (SLO) include reticular cells of which there are at least two different kinds, the follicular dendritic cell (FDC) and the fibroblastic reticular cell (FRC). Additional fibroblastoid cells are present in splenic red pulp and in the medullary sinuses of the lymph nodes (LN) (2). Cells of endothelial origin include blood endothelial cells of which there are both conventional “flat” forms as well as high endothelial venules (HEV) and the lymphatic endothelium that lines the afferent and efferent lymphatics, the subcapsular sinus, and the various lymphatic sinuses within the cortex, paracortex, and medulla (13). These structures are relatively stable in the resting state but undergo dynamic remodeling in the case of reactive LN. Reactive LN normally involute and presumably restore homeostasis once the activating trigger is resolved.

The lymphotoxin (LT) system, a member of the TNF family, is a key regulator of lymphoid architecture. For almost two decades now, the roles played by this pivotal system have been studied in the context of the development and maintenance of lymphoid reticular networks, HEVs, neo-angiogenesis, and lymphangiogenesis as well as their impact on immunological function. This pathway has been reviewed in detail (14–20). With a focus on recent advances, we highlight here the role of the LT system in the control of the reticular and endothelial elements within organized lymphoid structures.

## The Lymphotoxin System

**Figure 1:**
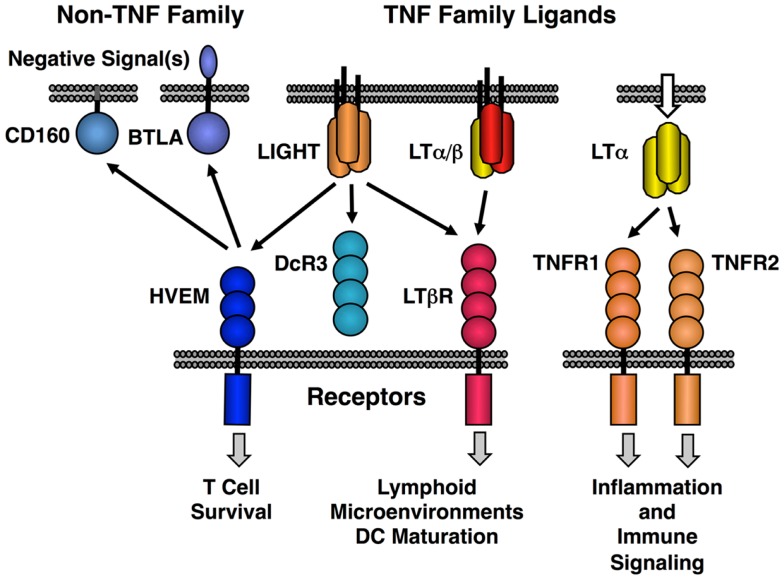
**Map of the interactions between LTβR, HVEM, and TNF receptors with the LT/LIGHT family and the Ig-super family members CD160 and BTLA**. LIGHT is shown in its membrane bound form, but it is also readily secreted like homotrimeric LTα.

The original LT protein, called LTα, is a member of the TNF family of ligands and is secreted as a homotrimeric molecule. Like TNF, LTα binds to the TNF receptors. An additional membrane bound form of LTα is composed of a heterotrimeric complex with a second protein called LTβ in a LTα1/β2 stoichiometry (LTα/β). LTα/β binds uniquely to the lymphotoxin-beta receptor (LTβR) (Figure 1). A second ligand, LIGHT (TNFSF14), binds to three different receptors; LTβR (TNFRSF3) with high affinity, another TNF family receptor called HVEM (TNFRSF14) and a soluble receptor named DcR3 (TNFRSF6B). LIGHT is found in both membrane tethered and secreted forms. Dimerization of LTβR by either LIGHT or LTα/β is sufficient to induce signal transduction (21, 22); however, it is likely that higher order oligomerization states of the receptor lead to either more intense or qualitatively different signaling (23). HVEM also interacts with several non-TNF family members, i.e., BTLA and CD160 and these proteins have context-specific regulatory effects on T and NK cell function (20, 24, 25). Human but not murine LTα can bind to HVEM and LTα binds to Troy (TNFRSF19) (26). However, these LTα interactions are low affinity and their physiological significance is questionable (27).

The soluble human LTα trimer appears to have specific functions. Most notably, LTα and LTβ knockout animals differ in that LTβ^−/−^ mice retain the mesenteric lymph node (mLN) while LTα^−/−^ mice lack all nodes (19). Likewise, the two LT knockout animals differ in their ability to survive cerebral malaria (28). The consequences of ectopic LTα gain-of-function in transgenic mice differ depending on whether or not LTβ is expressed with peripheral lymph node addressin (PNAd) display requiring LTβ (29). More recently, a specific role for LTα in the induction of gut IgA responses was defined (30). Indeed, differences between LTα and LTβ deficient mice have been attributed to additional and unique roles for the LTα homotrimer. Whether such LTα-specific functions are physiological can be questioned as the LTβ^−/−^ mouse represents potentially an LTα gain-of-function mouse. In a study using transfected cells, LTβ expression dramatically rerouted LTα expression into the membrane form to the detriment of secreted LTα (31). Thus, the loss of LTβ could initiate an LTα-driven inflammatory response especially in mucosal environments when TNF expression is minimal. In general, it is believed that the LTα/β heteromeric ligand represents a direct cell-to-cell communication system, whereas LTα3 is only secreted. In early studies, efficient secretion or shedding of the LTα/β complex was not observed (31–33); however, recently small amounts of shed LTα/β were detected in the blood and synovial fluid from rheumatoid arthritis patients using a highly sensitive assay (34).

The LTα/β-LTβR system has been implicated in many immunological events including maintenance of FDC networks, HEV, specialized macrophages capable of antigen capture in the splenic marginal zone and subcapsular sinus of the LN as well as dendritic cell (DC) homeostasis and differentiation (19, 35). This system also plays major roles in mucosal microenvironments with multiple consequences for epithelial cell biology and host defense/repair programs (36). LTβR signaling affects the state of specialized epithelial cells within the medulla of the thymus (37). The role of LT in epithelial cell interactions is beyond the scope of this review. Both LTβR and TNF-receptors activate canonical NFκB signaling, yet LTβR is distinguished by the efficient activation of the alternative NFκB pathway (19). Importantly, receptor internalization is required to shift NFκB activation from the classical to the alternative pathway and the nature of the ligand, e.g., soluble LIGHT vs. membrane bound forms of LIGHT or LTα/β may affect the ability to drive receptor internalization (38). The alternative pathway is typically linked to developmental and cell differentiation programs in contrast to the predominantly inflammatory role of the canonical pathway (39). The centerpiece of the alternate NFκB pathway is NIK kinase activation and NIK is required for many if not all of the developmental and differentiation events associated with LTβR (19).

Lymphotoxin-beta receptor is readily observed on most non-hematopoietic cells and on monocytic and DC lineages. Historically expression of the LT ligand was considered to be unique to hematopoietic lineages, especially B cells, activated T cells including Th1 and Th17 subsets, NK cells and innate lymphoid cells (40). This picture has become fuzzier of late, since LTβ RNA is readily observed in several other cell types such as in DC, hepatocytes, and hepatic oval cells (13, 41, 42). As LTβ RNA appears more abundant, LTα expression has been viewed as the limiting element in controlling membrane LTα/β display. It is less clear how often LTβ RNA reflects actual functional membrane LT, which requires expression of both LT proteins. Bona fide surface LTβ protein was observed on DC by histology and FACS; however, surface heteromeric LT α/β protein capable of binding LTβR actually has not been described outside of the hematopoietic system (43). As non-hematopoietic lineage cells and myeloid lineages usually express LTβR, it is theoretically possible that functional ligand produced within such a cell rapidly complexes with receptor, initiates signal transduction and the complex is processed. In such a case, cell autonomous LTβR signaling could be possible without actual transit to the surface and, moreover, surface ligand detection would be difficult. Furthermore, in cells with a high density of LTβR, there is evidence for autonomous signaling in the absence of any ligand (44).

The LT genes lie within a cluster with TNF in the class III region of the MHC and epigenetic control and chromatin organization are key elements of the regulation of cytokine expression within the cluster (45, 46). In general, understanding of the physiological transcriptional control of the LT and LIGHT genes remains incomplete. The LTβ promoter uses Ets1, Sp1, NFκB, and Egr-1/Sp1 transcription factors to drive RNA expression and expression was induced by TNF in a hepatocyte cell line (47). Furthermore, a regulatory element in exon 4 of the LTβ gene appears to interact with multiple sites within the TNF gene cluster (48). In general, LTα is expressed by activated lymphocytes and NFAT, NFκB, Sp1, and STAT elements are found within the promoter region. LTα expression can be induced by interleukin-12 p40 homodimer in myeloid lineages (49). Non-hematopoietic cells can also make LTα, for example, vascular smooth muscle cells secrete LTα in response to extracellular nucleotide activation of the P2Y_2_ receptor (50). Adding to this complexity, LTα RNA can be found in several variants. Interestingly, an alternate core promoter residing within the intron between exons 1 and 2 was predominantly utilized in T cells stimulated with TGFβ1 or FGF-7 (51). Clearly, a better understanding of the physiological regulation of the LT locus is needed.

## Lymphotoxin and the Differentiation States of Reticular Fibroblasts in Resting Lymphoid Tissues

**Figure 2:**
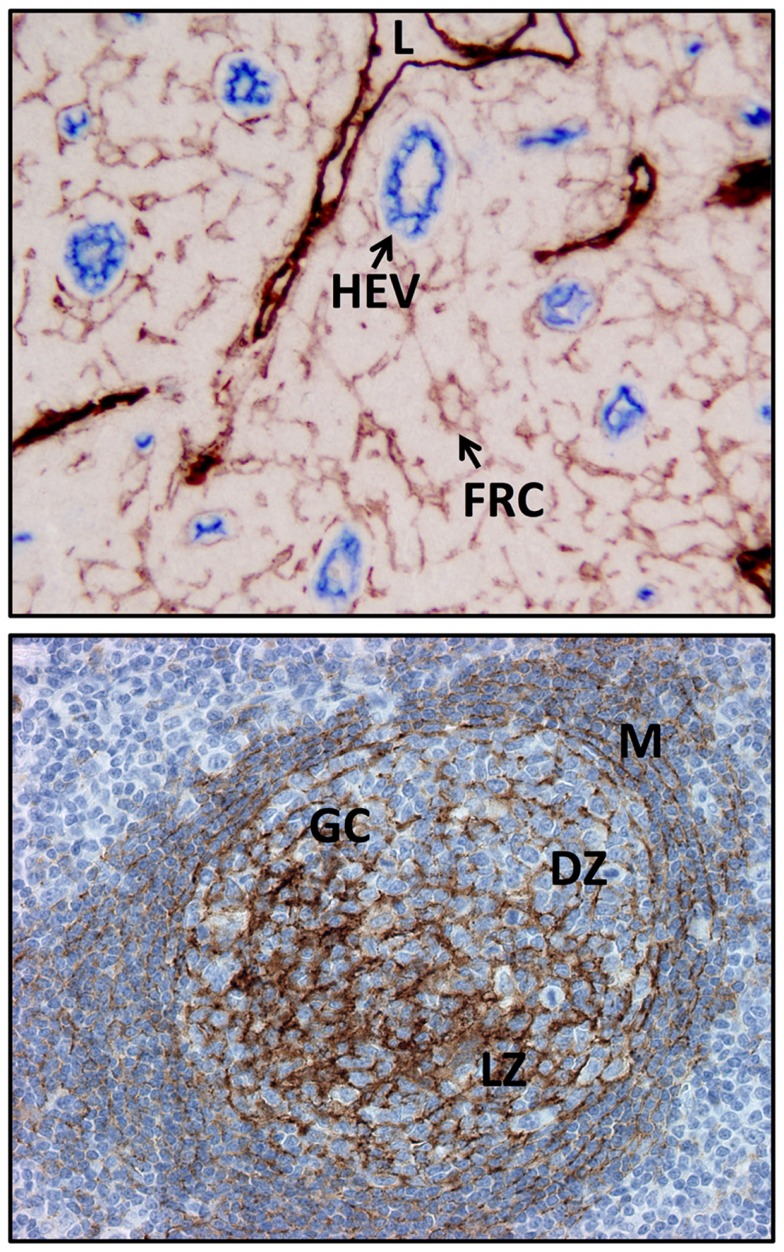
**Examples of FDC and FRC networks in the human tonsil**. Top panel shows the podoplanin positive FRC network and lymphatic vessels (both brown) and the surrounding HEV and non-HEV vessels (CD34, blue). Bottom panel shows the FDC network (CD35, brown) in a secondary follicle with a GC. Note light and dark (CD35 positive) regions are readily discerned along with the residual primary follicle or mantle zone surrounding the GC. L, lymphatic vessel; FRC, fibroblastic reticular cell, HEV, high endothelial venule; M, mantle zone or primary follicle; GC, germinal center or secondary follicle, DZ, dark zone; LZ, light zone.

The cells forming the lymphoid reticular scaffolds are of mesenchymal lineage and generally are grouped into two broad categories based on their locations within organized lymphoid organs (Figure 2). FDC scaffold the B cell follicles and perform a multitude of functions related to antigen-presentation, cell survival, and handling of apoptotic cells (9–12). They possess a unique recycling mechanism to protect captured antigen from degradation and retain it for long-term presentation (52). FDC express the chemokine CXCL13 required for follicular compartmentalization of B cells (10). The second major category of reticular networks encompasses the T cell region of both LN and the spleen and is formed by FRC (2, 5, 8). FRC have multiple functions including the attraction of CCR7-expressing T cells and DC by expression of CCL19 and CCL21, T cell survival signals, and formation of collagen fibers. As such, they are fundamentally different from the FDC networks. A third category called a marginal reticular cell (MRC) has received recent attention. This cell sits under the subcapsular sinus close to B cell follicles, combines elements of both FDC and FRC and has been proposed to be a progenitor cell to the two major lineages (5, 53). Within the FRCs, there is heterogeneity, for example, VEGF-expressing FRC are enriched in vessel-rich areas (54). Also, using a lineage-tracing system for past expression of CD21, a population of mesenchymal cells in the T zone was identified that are similar to FRCs, yet express higher levels of TGFβ and lower amounts of CCL19 and CCL21 relative to FRCs (55). How these cells are related in lineage to the classical FRCs remains to be determined.

To understand reticular cell differentiation pathways, it is important to appreciate the embryological underpinnings of LN development (16). Early LN anlagen are formed from buds of endothelial and mesenchymal tissue. The mesenchymal cells progress through an ICAM1/VCAM1+ intermediate phenotype and at this stage, upon LTβR activation by hematopoietic lymphoid tissue inducer (LTi) cells, they acquire an ICAM1/VCAM1/MAdCAM1+ stromal organizer phenotype (56). This cell is often referred to as a lymphoid tissue organizer (LTo). The LTo cells now express CCL19, CCL21, CXCL13, IL-7, and RelB, and these markers will typify future specialized stromal elements in the mature organ. The reliance on LTβR signaling at the stromal organizer switching point is believed to account for the impaired development of LN in mice deficient in components of the LT pathway. Importantly, ontogeny has provided an important guide in understanding lymphoid tissue changes as embryological paradigms are largely recapitulated during inflammation (57, 58). Studies on the role of the LT system have always been plagued by the complexity of the direct knockout animals, i.e., the lack of LN and the issues of dissociating embryological programs including postnatal events from physiological maintenance/homeostasis. Historically, pharmacological inhibition of LTβR signaling has been the most direct method to achieve dissection, however, cell-specific or inducible genetic manipulations now provide powerful tools.

### Reticular network progenitors

**Figure 3:**
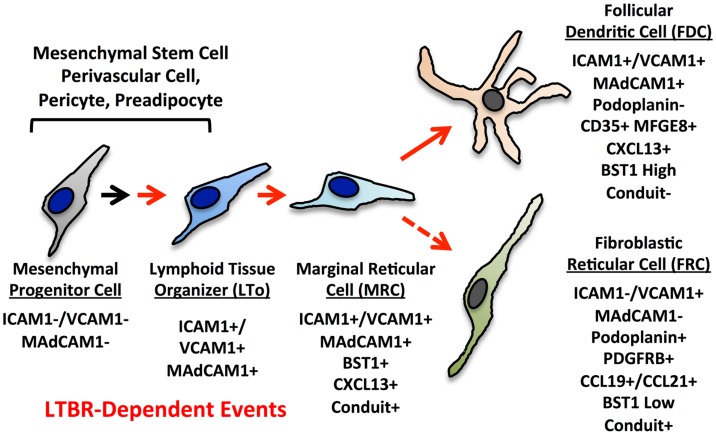
**Putative differentiation pathways for the FDC and FRC networks showing major phenotypic changes**. The MRC is speculated to be a progenitor for FRC and FDC lineages based on phenotype, but this relationship has not been formally proven.

The current paradigms for reticular cell specialization in the SLO invoke an early progenitor cell that is somewhat similar to a mesenchymal stem cell (MSC) (Figure 3) (6). Signals early in development provided by retinoic acid or RET ligand depending on the context lead to an immature organizer cell (16). In most contexts, LTβR signaling is required for progression to the mature organizer or LTo that releases chemokines and displays surface adhesion molecules to nucleate the emerging LN anlage. Brendolan and colleagues recently were able to use lineage-tracing methods to map this pathway using the transcription factors Nkx2–5 and Islet1 (59). The Nkx2–5/Islet1 cells differentiated into all three major reticular lineages, MRC, FDC, and FRC as well as into classical mural cells or pericytes, but not the endothelial lineages.

### FDC networks

Lymphotoxin-beta receptor signaling is essential for the development, differentiation, and maintenance of FDC networks in the SLO, in mucosal compartments and in chronically inflamed sites, i.e., so-called tertiary lymphoid tissues/organs (TLTs/TLOs) (9–12). TNF signaling is also critical for the differentiation of the FDC phenotype, but not FDC maintenance at least in a secondary follicle. Surface LT on B cells is crucial for the development and maintenance of the FDC networks in the spleen as shown by the use of classical bone marrow chimeras and more specifically with a conditional B cell selective knockout of LTβ (60–63).

Historically, the term “dendritic cell” in this setting was an unfortunate misnomer and contributed to the confusion surrounding their true origin. Obscuring their origin even further, FDC “precursors” could be transferred from bone marrow; however, this observation preceded the discovery of MSCs in the bone marrow. In a breakthrough, the precursor cells to FDCs were recently identified as perivascular cells resembling pericytes (64). Pericytes are members of a subset of fibroblastoid cell types including classical pericytes also called mural cells, vascular smooth muscle cells, adventitial fibroblastoid cells, and stellate cells (hepatic, pancreatic, pituitary, astrocytes, etc.). MSCs are progenitors of both this immediate “pericyte” family as well as a much wider group of cell types including adipocytes, chondrocytes, osteoblasts, fibroblasts, myofibroblasts, synoviocytes, and podocytes. The exact differences between MSC and perivascular cells or pericyte progenitors remain murky (65, 66). There is some semantic confusion as a pure classical definition of pericytes would include direct contact with an endothelial cell without interruption by a basement membrane. In practice, lacking unique cellular markers, there appears to be a spectrum of related perivascular cells.

Aguzzi and colleagues used multiple approaches to understand the origin of FDCs. They were able to lineage map PDGFRβ+ precursors into FDC and ablation of these same cells led to the loss of FDC. Additionally, transfer of adipose-derived perivascular cells into the renal capsule resulted in the formation of a local FDC network (64). B cells were the sources of LT and TNF for FDC development, yet in the absence of B cells, LTi cells were implicated as a key source of LT. It should be noted that these approaches were not completely definitive, e.g., the markers PDGFRB and MFGE8 are not specific for pericytes and FDC, respectively. This limitation in the study highlights how the pericyte field suffers from the lack of absolutely unique markers (67). Nonetheless, PDGFRβ positive cells in adipose tissue are generally accepted to be a pericyte/MSC and these cells were able to give rise to FDCs. Therefore, the differentiation of mesenchymal progenitor cells into a reticular cell that is highly honed to retain antigen within the follicular microenvironment, requires either LT or TNF signaling provided by local lymphoid cells. This system provides an excellent example of the ability of the lymphoid cells to locally shape mesenchymal lineages.

Established FDC networks exist in both a primary form, i.e., in a follicle lacking a germinal center (GC) reaction or in GC containing secondary follicles wherein the network is further differentiated (10, 68). Maintenance of established networks absolutely requires LTβR signaling as shown by pharmacological inhibition of the pathway (69–73). Mice genetically deficient in elements of the LT system have been instrumental in defining the roles of splenic FDC. For example, spleens from LT-deficient mice displayed vastly reduced CXCL13 expression and pharmacological inhibition gave roughly similar results (74). Using various LT pathway deficient mice or bone marrow chimeric mice, lack of FDC resulted in profound impairment in both class switch recombination and affinity maturation. These results were probably complicated by additional defects in the splenic marginal zone, trafficking of cells to the correct compartments, and DC biology, etc. (14, 75). For example, the impressive loss of immune complex trapping on splenic FDCs in monkeys following LTβR inhibition was potentially a combination of both degradation of antigen trapping machinery in the primate marginal zone as well as FDC collapse (71).

The impact of FDC disruption has been studied by many approaches, but a selective depletion system described recently by Cyster and colleagues provided a relatively clean analysis (76). Loss of FDC networks collapsed follicular organization, reduced CXCL13 chemokine production and led to abortive GC reactions probably due to lack of long-term antigen retention (76). In monkeys, splenic FDC networks were substantially collapsed after only 1 month of pharmacological inhibition of LTβR signaling (71). Curiously, GC reactions in the mLN may be less dependent on proper FDC networks than in the spleen or peripheral LN (pLN) (76). mLN are clearly different from pLN in terms of their developmental requirements (76–79). In general, the role of LTβR signaling in FDC maintenance during a GC reaction remains poorly described.

### FRC networks

Fibroblastic reticular cell are reticular cells that scaffold the T cell rich regions of organized lymphoid structures (1, 2, 4, 7, 8). The chemokines CCL19 and CCL21 are produced by FRC and orchestrate the movement and attachment of lymphocytes along this backbone with DCs being more tightly attached and T cells loosely marching along the network to spatially facilitate T-DC encounters. Clean experiments to dissect immune function in the presence and absence of FRC are lacking and indeed some have questioned how much the FRC actually facilitates encounters (80). FRC also provide the IL-7 for T cell survival and the control of homeostatic compartment size in a manner reminiscent of BAFF and B cell survival (81). FRC have the capacity to directly present antigen to promote peripheral tolerance and they can respond with nitric oxide production during acute inflammation thereby dampening T cell responses (82–84). The scaffold itself is composed of collagen fibers that are made and wrapped by the FRC, forming a functional unit called a “conduit” (85). These conduits are found in all lymphoid tissues, e.g., spleen, thymus, LN, and TLT and appear intrinsic to an organized microenvironment. The conduits allow for very fast transport of low molecular weight proteins, i.e., up to roughly 70 kDa from the lymph or blood to the LN parenchyma. DC residing on the FRC can sample the conduit contents, raising the potential for direct detection of small antigens. Additionally, it is reasonable to propose that conduits allow the LN to sense chemokine/cytokine signals emanating from an inflamed tissue bed.

Recently, three studies have contributed greatly to understanding FRC differentiation. First, the Turley group in conjunction with the Immunological Genome project purified and analyzed the transcriptomes of FRC, blood endothelial, and lymphatic endothelial cells along with a fourth so-called double-negative cell (DNC) population that was negative for the endothelial marker CD31 and the FRC marker podoplanin (86). The DNC population was pericyte-like and most closely resembled FRC. This work provided a unique molecular fingerprint of these stromal populations and suggested a lineage relationship between pericytes and FRC. Caamano and colleagues showed that embryonic progenitor cells resembling preadipocytes can differentiate into lymphoid stromal cells (87). LTβR signaling, via the alternate NFκB pathway, was required to stop a progenitor cell from embarking on an adipocyte program and to direct a shift toward a lymphoid stromal cell differentiation pathway. This capacity was demonstrated using both embryonic and adult progenitors derived from adipose tissue. Moreover, they hypothesized that adipose tissue is a source of lymphoid stromal cells. Consistent with these observations, LIGHT-LTβR interactions can inhibit the program of adipocyte differentiation (88, 89). Third, Ludewig and coworkers exploited a unique CCL19-Cre mouse with expression limited to the FRC (90). Using this tool, LTβR could be selectively deleted from FRC-like cells. Surprisingly, these mice developed LN and FRC/conduits normally; however, the LTβR-deficient FRC displayed reduced CCL19, CCL21 chemokine, and podoplanin expression. It is likely that insufficient CCL19 driven Cre is expressed at the early progenitor phase to remove LTβR prior to LTo generation and hence LN development proceeds normally. The mice bearing LTβR-deficient FRC poorly managed a murine herpes virus infection suggesting defective FRC diminished the immune response. These studies indicate that some aspects of FRC are under LT control, both developmentally and upon ectopic relocation, i.e., a setting similar to the induction of TLT.

Therefore, it appears that, like FDC, *de novo* development of FRC requires LTβR signaling. This result was best illustrated in the earlier observations on the formation of FRC networks in the spleen and TLT (74, 91). Despite these elegant studies, the basic question is less clear as to whether established FRC networks in either resting or reactive LN are under continuous LTβR control as observed with FDC. In prior analyses by the Cyster group, there was a clear developmental dependence on LT positive B cells for the splenic T zone FRC and their expression of CCL21 (74, 92). The development of splenic FRC, like FDC, continues from birth out for several weeks and indeed neonatal inhibition of LTβR signaling reduced CCL21 levels (a surrogate for FRC maturation) (92, 93). However, neither pharmacological inhibition of LTβR in adult mice nor transfer of LT-deficient lymphocytes appreciably reduced CCL21 levels in either spleen or mLN (74, 92). As in the spleen, FRC in LN continue to develop for 2–6 weeks post gestation based on CCL19 expression (90). The loss of LTβR on FRC using the CCL19-Cre system caused major disruptions in the nature of the FRC network (90). Likewise, combined TNF and LTβR signaling was synergistic leading to generation of collagen containing conduit-like fibrils in cultures of LN-derived stromal cells (94). One interpretation of this result is that canonical NFκB signaling by TNF is required to resupply the components to drive long-term alternate NFκB signaling by LTβR. In the light of these various observations, it is perhaps perplexing that pharmacological LTβR inhibition in adult mice did not affect CCL21 levels. However, these observations are consistent with a developmental role for LTβR signaling in FRC development, but not in the maintenance of established networks. Further histological analysis of non-matrix markers such as podoplanin, PDGFRβ, etc., expression in FRC networks following LTβR-Ig treatment need more careful examination. Therefore, in contrast to FRC development and FDC networks, the existing data indicate that the maintenance of established FRC networks is not under LT control.

## The Role of the Lymphotoxin System in Regulation of the Resting Lymphoid Endothelium

### High endothelial venules

A prominent feature of LN is the presence of specialized post-capillary venules called HEVs that allow for the transit of lymphocytes from the blood into the LN parenchyma (13). The selective display of various adhesion molecules, coupled with specific chemokine triggers for integrin activation differentially gate access to the unique microenvironments of the skin, mucosa, and various LN. The robust transit of lymphocytes through the endothelium presses the endothelial cells into plump shapes giving the venule its characteristic “high” status (95). Development and maintenance of the HEV specifically requires LTβR signaling via the alternative NFκB pathway with a dialog occurring between LT positive DC and LTβR positive endothelial cells to promote the “HEV program” (96–98). The role of LTβR in HEV maintenance was conclusively demonstrated by pharmacological intervention of LTβR signaling in adult mice (99, 100). Developmentally, the requirement for LTβR signaling was less obvious since LN development itself is arrested in knockout mice, although addressin expression was reduced both in the mLN in LTβ deficient mice and in the occasional LN that develop following gestational inhibition of LTβR signaling (101, 102). Recently, deletion of LTβR expression selectively in endothelial lineages curtailed development of a normal repertoire of LN’s, yet HEV development was blocked in remaining rudimentary LN (103). This study proved that the HEV program was directly under LTβR control and not a consequence of more indirect events related to a disrupted lymphoid architecture. Although some HEV are also associated with the pericyte-like DNCs (86), FRC appear to extend directly to the HEV, and wrap the abluminal face (104). It is conceivable that the FRC/HEV acts as a unit comparable to the astrocyte/pericyte/endothelial cell neurovascular unit or the relationship between stellate cells, pericytes. and endothelial cells in the liver sinusoids.

The PNAd molecule on HEV is a complex sulfated glycan assembled on specific scaffold proteins in both N- and O-linked forms. PNAd binds to L-selectin on the lymphocyte triggering the initial rolling. The basic LTβR-controlled “HEV program” encompasses the direct induction of scaffold gene expression in LN such as MAdCAM1, GLyCAM1, and CD34, as well as the biosynthetic machinery needed to assemble on the scaffold proteins the unique sulfated glycans that bind to L-selectin (13). MAdCAM1 is an addressin for the integrin α4β7, as well as itself being a scaffold for PNAd attachment. Vascular MAdCAM1 expression in the mLN is LTβR-dependent, although inflammatory signals such as TNF or secreted LTα can also induce MAdCAM1 expression (13). MAdCAM1 expression on the sinus floor of the splenic marginal zone and on FDCs is also LTβR-dependent as is mostly likely the expression on MRCs and the LN subcapsular sinus (105). Vascular MAdCAM1 is elevated in colitis in an LTβR-dependent manner; however, it is unresolved whether this component is indirect and secondary to effects on the disease processes (14).

HEV can readily accumulate radiolabeled sulfate and contain elevated levels of the machinery involved in sulfate uptake and transport (106, 107). In addition to specific sulfated glycan that comprise PNAd, sulfation is also involved in chemokine retention. Heparin sulfate is crucial for endothelial chemokine binding and lymphocyte trafficking as well as DC recruitment to the lymphatics (108). Whether there is any dependence on LTβR signaling for endothelial sulfate capture or sulfated matrix components has not been explored.

While considerable progress has been made uncovering the role of the LT pathway in lymphoid vascular biology, there remain murky aspects of HEV development and specialization within unique environments, e.g., mucosal, peripheral, TLT, tumor, etc. For example, the homeodomain transcription factor Nkx2.3 controls vascular compartmentalization in the spleen yet its deletion led to the formation of splenic HEV-like vasculature complete with expression of PNAd and CCL21 (109). HEV formation remained LTβR-dependent in line with the appearance of splenic PNAd positive structures in a mouse with a constitutive gain in alternative NFκB signaling (110). Nkx2.3 deficiency also led to altered splenic FRC structures and the presence of LYVE1 positive lymphatic-like structures thus pivoting the spleen toward an “LN architecture” (111).

### Lymphatics

The afferent lymphatics transport cells and soluble substances from the draining tissue to the subcapsular sinus. From the subcapsular sinus, DC find their way into the T zone parenchyma while T cells either flush through or they can enter the LN parenchyma via peripheral medullary sinuses (112). Recirculating lymphocytes leave the LN parenchyma by entering cortical sinuses that feed into medullary sinuses and efferent lymphatics (113). The lymphatic endothelial cells are an important source of sphingosine-1-phosphate that elicits lymphocytes to leave the parenchyma and enter the sinuses (114). Whether LTβR signaling is crucial for lymphatic function is less clear, although defects in lymphatic function were observed in LT-deficient mice (115).

## Lymphotoxin Pathway and Adaptation in the Reactive State

During immune responses, the reticular networks and the endothelium undergo growth and remodeling with an overall increase in LN cellularity. Such enlarged LN are termed “reactive” and normally the LN involute and return to the resting state once the triggering stimulus is resolved. Experimentally, reactive LN are induced by immunization with an adjuvant such as alum, complete Freund’s adjuvant (CFA) or Montanide, or by infection with a pathogen. Total LN cellularity in both the resting and hypertrophic reactive state is LTβR-dependent (99, 116, 117). Whether the stroma or vasculature bears an imprint of prior reactivity is unknown, although gross lymphangiogenesis following inflammation is certainly reversible (118).

### Reticular network

Within the T zone, the reticular network expands with the enlarging T zone and the stromal cells undergoing proliferative expansion (119–121). The concentration of reticular fibrils under the follicles where T cells frequently interact with DC is known as the cortical ridge and this ridge becomes more prominent after immunization with ovalbumin (OVA) in alum (119). Following immunization with OVA in CFA, the stromal cells undergo an initial proliferative burst between days 0 and 2 that is dependent on CD11c+ DC cells and independent of lymphocytes. This burst is followed by continued proliferation and expansion in cell numbers that is detectable by day 5. The expansion was abrogated in the absence of lymphocytes and B cells contributed to the full expansion, while T cells contributed to the continued high proliferation rate (120). Recently, Luther and colleagues carefully analyzed changes in the FRC network following immunization with OVA in the adjuvant Montanide in mice containing transferred OVA specific T cells (121). Similar to changes induced with OVA/CFA, OVA/Montanide-induced a rapid CD11c+ cell-dependent stromal proliferation, and modest stromal expansion could be detected within 40 h. Stromal cells continued to proliferate and expand, and the expansion by day 5.5–6 was abrogated in RAG2^−/−^ mice. Significantly, this study revealed that LTβR-Ig treatment did not affect the modest expansion at day 3, but did reduce FRC expansion at day 5.5–6 suggesting a role for LTα/β expressed by lymphocytes. A number of genes were previously shown to be altered in activated FRC and in this recent study, these cells displayed elevated levels of podoplanin and smooth muscle actin (86, 121). While maintenance of the resting FRC is not obviously under LTβR control, it is clear that events occurring in reactive FRC are LT/LIGHT-dependent and this dependence may underlie partially the profound effects of LTβR-Ig treatment on reactive LN. Again, the reticular changes occurring during inflammation appear to recapitulate programs that are utilized during development.

As hinted above, reticular networks may differ in their response to various immunological challenges including acute vs. chronic settings. For example, in contrast to Alum, CFA as an adjuvant leads to disruption of normal ER-TR7 patterning (ER-TR7 is a marker of collagen fibrils that is usually a surrogate for FRC presence), with loss of distinct B and T zones (119). A more detailed analysis of the time course is needed to determine whether the distinctions between Alum and CFA may in part reflect differences in kinetics. During a viral response, such as with LCMV infection, CCL21 and CXCL13 chemokine expression is downregulated during the first 8 days in the spleen and this response was partly dependent on interferon-γ but was minimally impacted by LTβR-Ig (119, 122, 123). The same process occurs in draining LN (123). Although, the LCMV clone-13 strain can infect the FRCs, this phenomenon of chemokine downregulation also occurred with OVA/LPS suggesting that this is a general phenomenon in inflamed lymphoid tissues (123). The loss of chemokine expression with viral infection may be in part attributable to loss of stromal cells (122). After this initial viral-induced stromal disruption, stromal cells recover and lymphoid organization is gradually restored. It is this process of recovery that is accelerated by LTi cells and is at least partially dependent on LTβR (122).

During LN hypertrophy, some of the most notable changes occur in the medulla (121, 124). This compartment normally appears condensed at homeostasis, but upon immune stimulation, the region swells and fills with lymphocytes. By day 6 after NP-OVA/CFA immunization, the parenchyma is enlarged relative to the area covered by the lymphatic marker LYVE1, and areas of collagen IV reticulum-rich and reticulum-poor areas can be identified. B cells are preferentially localized to the reticulum-poor area and newly generated plasma cells localize to the reticulum-rich areas. The significance of this compartmentalization remains to be elucidated. Medullary remodeling was reduced by LTβR-Ig treatment in a manner that appeared independent of its effects on FDC and HEV (124).

### Vasculature

Following immunization, the vasculature also undergoes proliferative expansion that is accompanied by major phenotypic changes in the HEV. HEV, non-HEV blood endothelial cells, and lymphatic endothelial cells all proliferate and expand coordinately with remodeling of the feeding arteriole to deliver more blood flow (100, 120, 125–128). Similar to stromal cell growth, endothelial cells in LNs stimulated with OVA/CFA undergo initial CD11c+ cell-dependent proliferation followed by lymphocyte-dependent expansion (120, 125). HEVs can be seen to grow in length, width, and branching as imaged using optical projection tomography (116, 129). The HEV expansion triggered by LCMV infection is sensitive to LTβR-Ig and partially dependent upon B cell-derived LT. As B cells are not required for HEV maintenance at homeostasis (99, 100), this role of B cell-derived LTβ appears to be specific to the inflamed LN. With inflammation, there is the phenomenon of venularization whereby adjacent endothelium adopts a post-capillary venule phenotype (130). In inflamed LN, this change seems to occur as HEV (i.e., PNAd+) endothelial cells expand to a greater degree than PNAd-blood endothelial cells (120). One unresolved question is whether the reduction of HEV expansion in the absence of LTβR signaling reflects inhibition of HEV differentiation or inhibition of HEV proliferation? Anderson and colleagues observed that endothelial cell proliferation occurred frequently at transitions between high and flat endothelium, consistent with simultaneous proliferation and differentiation (131). Another area that is still poorly understood is whether the LTβR-dependent HEV expansion reflects LTβR function in reticular cells, DC, or the endothelial cells.

In the reactive LN, numbers of both blood vessels and lymphatic sinuses increase and this expansion is accompanied by lymphatic endothelial cell proliferation (100, 120, 126). The increase in the number of lymphatic structures was partly sensitive to LTβR-Ig (100), but it is unknown whether LTβR-Ig blocked proliferation or impacted other facets of lymphatic growth. Somewhat surprisingly, LTβ^−/−^ mice showed greater lymphangiogenesis and angiogenesis in inflamed lung and skin and hence, the general role of LTβR in LN vascular growth remains ill-defined (115). In contrast, LTα^−/−^ mice showed reduced lymphatic function and reduced lymphangiogenesis with skin inflammation, and transgenic LTα expression drove lymphangiogenesis, suggesting a pro-lymphangiogenic role for LTα3 as well as a role for LTαβ in “sequestering” LTα subunits. In agreement with a role for LTα3, TNF inhibition or TNFR1 deficiency were shown to partially reduce lymphangiogenesis in lung (132).

**Figure 4:**
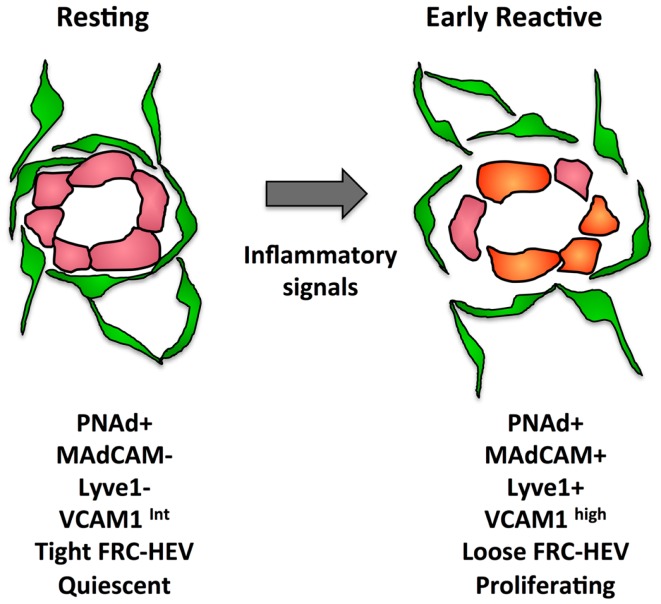
**Illustration of some of the changes that occur in the FRC-vascular units during the resting to reactive transition in a lymph node**.

In addition to proliferative expansion, the HEVs also undergo phenotypic alterations. The phenotypic changes seen in reactive HEV are illustrated in Figure 4. At day 1 after injection of bone marrow derived DCs or OVA/CFA immunization, VCAM1 is upregulated and there is a greater trafficking of lymphocytes inwards via HEVs (104). This early change corresponds to disruption of the tight sheath of FRCs around vessels and the HEV activation is similar to the early ICAM1 upregulation seen after fever-range thermal stress (104, 133). By day 4 after oxazalone stimulation, HEV endothelial cells show reduced expression of proteins associated with the mature HEV program, i.e., LTβR, the scaffold protein GlyCAM1, and the sulfo-transferase HEC-GlcNAc6ST (100, 134). The downregulation of these HEV program proteins was accompanied by upregulation of MADCAM1 perhaps reflecting an immature HEV, since MADCAM1 is normally only expressed in peripheral nodes during development and in mucosal lymphoid tissues (100, 135). In the same time frame, LTβR expression decreases and PNAd and LYVE1 were colocalized despite LYVE1 being a typical lymphatic marker (100). It was unclear from this study whether this colocalization resulted from lymphatics that expressed PNAd or HEVs that expressed LYVE1. However, vessels with the morphology of HEVs in non-Hodgkins lymphoma patients expressed LYVE1 and blood vessels during development can express LYVE1, suggesting that the colocalization of PNAd with LYVE1 represents HEVs that have upregulated LYVE1 (136, 137). This PNAd/LYVE1 colocalization at day 4 was blocked by LTβR-Ig treatment at day 0 (100). By day 7, the mature HEV markers began to rebound while MADCAM1 and LYVE1 regressed, and this rebound phase was dependent on B cells and also sensitive to LTβR-Ig.

The reprograming of the HEV differentiation state as a LN enters the reactive state was noted to be similar to changes occurring after surgical interruption of afferent lymphatic flow (99, 100, 138). Consistent with this notion, Ruddle and colleagues showed a transient reduction in LN accumulation of subcutaneously injected Evans blue and skin-derived DC in the first 4 days after the onset of inflammation, which was followed by increased lymphatic flow (100). In contrast, Randolph and colleagues showed increased LN accumulation of skin-derived DC in the same time frame (126). These studies both applied FITC epicutaneously in pre-immunized mice and then tracked FITC+ DC, and it remains unresolved whether the differing results reflected different preimmunization strategies (oxazalone application vs. KLH/CFA injection) or different kinetics of these strategies. In human subjects, Braathen and colleagues measured lymphatic flow via cannulation of a dermal lymphatic, and found that application of a skin irritant resulted in a transient reduction in dermal lymphatic drainage before the drainage increased to above baseline (139). Whether the transient decrease was related to the trauma of skin cannulation 2 days prior is a caveat in interpreting the findings, although the trauma itself could be taken as a form of immune stimulation. However, with regard to the idea of HEV reprograming, the basic biological event that underlies this phenomenon remains a question, i.e., is it the disrupted flow, the inflammation or a dilution of LTβR signaling due to loss of ligand and/or receptor? Do the HEV in the inflamed LN need transport of soluble factors or cells via the lymphatics to prevent the reprograming?

## Putting it Together in the Reactive Node: Coordinate Regulation of Vasculature and Stromal Alterations and the Role for LTβR Signaling

**Figure 5:**
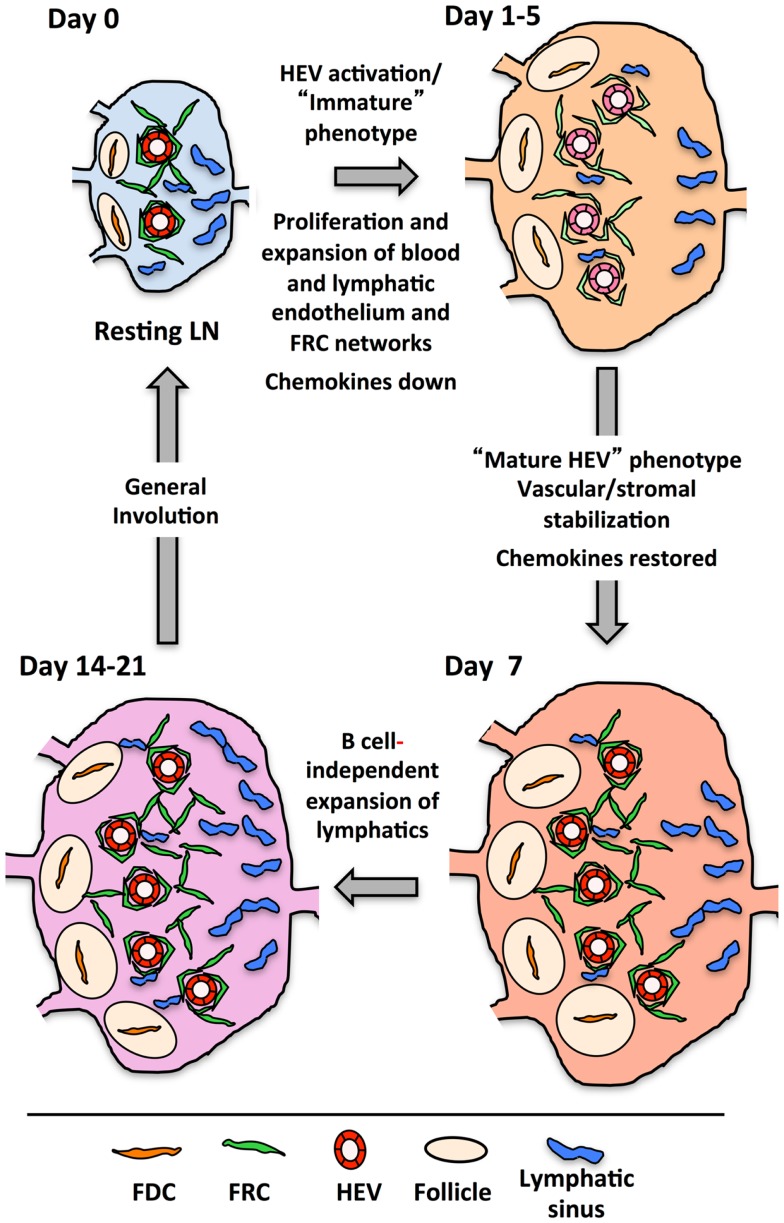
**Drawing shows the cycle that lymph nodes transit from resting to reactivity with some of the changes occurring at each stage**. The actual timing of each stage will depend on the nature of the reactive trigger, e.g., immune complex, innate stimuli, virus, etc., and its subsequent clearance.

Figure 5 presents a generalized view of some of the changes occurring in the LN as it cycles between resting and reactivity. In the first 2 days following immunization with OVA/CFA or bone marrow derived DCs, there is a lymphocyte-independent initiation phase marked by endothelial and reticular cell proliferation with parallel VCAM1 upregulation on HEV. From days 3–5, there is an expansion phase whereby endothelial cells and stromal cells continue to show high levels of proliferation and now noticeably expand in number. This expansion in most cases is at least partly dependent upon B cells and T cells contribute partially to the continued high level of endothelial and stromal cell proliferation during this phase (100, 120, 121, 124–126). The expansion phase correlates well with the morphologic HEV expansion that is dependent on B cell-derived LTβ and the LTβR-Ig sensitive OVA/Montanide-induced FRC expansion (100, 116, 121). In this context, the upregulation of MADCAM1 and downmodulation of GlyCAM1 and HEV-GlcNA6ST observed by day 4 and could potentially represent a reinstatement of the HEV programing that typically occurs during early LN anlage development either in early activated HEV cells or in newly expanded cells (100). Assuming that the colocalization of LYVE1 with PNAd by day 4 represents upregulation on HEV cells, consideration of the potential significance of LYVE1 alterations may provide some clues. LYVE1 on developing skin collecting lymphatic vessels is downregulated with the recruitment of smooth muscle cells (140), correlating vascular destabilization/immaturity with LYVE1 expression. During the initiation phase, the tight, presumably stabilizing sheath of FRCs around the vessels is disrupted and vascular permeability is increased (104, 131). It is conceivable that upregulation of LYVE1 on HEV cells, like VCAM1 upregulation is an early activation marker that reflects a state of HEV destabilization. Taking the LYVE1 upregulation as a marker of early HEV activation, then, both early HEV activation as well as subsequent expansion appears to be dependent on LTβR, and we would predict that the MADCAM1, GlyCAM1, and HEC-GlcNA6ST alterations are also LTβR-dependent.

The disrupted organization of FRCs around vessels during the initiation phase probably reflects a more general reticular network disorganization that was seen with OVA/CFA or, with a mild delay, with viral infection. The viral-induced stromal dysfunction was minimally sensitive to LTβR-Ig (123) suggesting that the reticular network disruption may be relatively LTβR-independent. Thus, early vascular and stromal changes in the reactive node may be differentially regulated by LTβR signals. This is in contrast to the later, LTβR-dependent reticular remodeling.

After the expansion phase, the vasculature undergoes a phase wherein quiescence and stabilization are re-established. By day 7, mature HEV markers rebound, robust endothelial cell proliferation slows and FRCs re-organize more tightly around the vessels (100, 104). Thus, restoration of mature HEV markers parallels the re-establishment of vascular quiescence/stabilization and stromal integrity. The LTβR-dependent stromal recovery after viral infection that was studied in the spleen may correspond to this phase in the LN, and, if so, would further support the idea of a global vascular-stromal re-stabilization. Whether, LTβR signals are needed for all the vascular changes and how the medullary remodeling is connected is unknown.

Following the vascular quiescence and stabilization phase, there is additional growth, at least in the lymphatic vasculature as well as considerable medullary remodeling (100, 124, 141). Lymphatic expansion is B cell dependent up to at least day 7, but by day 14, lymphangiogenesis is occurring in the absence of B cells (100). This late lymphangiogenesis coincides with expansion of cortical and medullary lymphatic sinus relative to that of subcapsular sinuses, which expanded at an earlier time point (141). This observation suggests that some cortical and medullary expansion is B cell-independent. LTβR signaling appears to be important for medullary remodeling, but the actual specifics of which events are being regulated needs further investigation. Any role for the LT pathway in the restoration of homeostasis or the “involution phase” is also unexplored.

In conclusion, LTβR signals in the reactive LN are needed for multiple phases of vascular and stromal alterations, yet major elements of this picture remain out-of-focus. How exactly does LTβR control the initial growth and phenotypic alterations? Do these requirements reflect homeostatic functions of LTβR or are these new functions in the context of an inflamed LN? Fu and coworkers recently showed that LIGHT-LTβR signaling was important for the growth of reactive nodes but not for homeostatic LN cellularity suggesting that additional LTβR ligands and functions are engaged in the reactive setting (117). Lastly, does the rebound of mature HEV markers and the stromal re-organization indicate an increased level of LTβR signaling during the phase of re-established quiescence and stabilization?

## LTβR and Tissue Remodeling in Disease

### Chronic reactivity in lymph nodes

Chronic LN reactivity can change lymphoid architecture and this phenomenon has been well described especially in the context of viral infection (2, 3, 142). In primates, both HIV and SIV infection led to altered FRC function and a lack of T cell survival support. Interestingly, the chronic reactivity in HIV infected LN culminates in fibrosis perhaps mimicking aspects of other fibrotic diseases (143). This observation is consistent with increased smooth muscle actin expression in the FRC in reactive murine LN (121). Reactive LNs, hyperplasia, lymphadenopathy, and probably imbalanced reticular networks often characterize autoimmune disease (144). For example, abnormal FDC networks were noted in some SLE patients, albeit there is considerable heterogeneity in this population (145, 146). Additionally in rodent models of lupus, FDC networks are altered in the MRL.lpr mouse (147). Aging is also associated with some degradation of lymphoid architecture (148). In most of these cases, the relationship to potentially impoverished LTβR signaling is unknown. Given the need for organized lymphoid architecture in a well-functioning immune system, this remains an important area for further investigation.

### Tertiary lymphoid tissues

Chronic inflammation drives the formation of semi-organized lymphoid structures in basically all organ settings including lungs, heart, stomach, intestine, kidney, CNS, glands, skin, joints, and vasculature (149–153). These TLT are also present physiologically in the gut where they develop upon colonization with the microbiome (154). A wide spectrum of structures can be observed ranging from relatively poorly organized perivascular aggregates to more complete LN-like structures that display HEV development, T/B cell segregation, GC formation, and the presence of specialized FDC and FRC reticular networks (152, 153). For example mature TLT are found in salivary glands in female NOD mice, yet a B cell dominated structure lacking FDC networks is observed in the young male NOD lacrimal glands (155, 156). TLT can accompany Th1 and Th17 driven responses and even assemble next to classical *Mycobacterium*-driven granulomas (157–159). Notably, TLT are induced by ectopic expression of LTα or combined LTα and LTβ in multiple organ systems and these studies have provided considerable insight into the biology of these structures (42, 149, 160). There appear to be multiple routes to TLT formation including those induced by RORγt positive cells such as type III innate lymphoid cells (ILC-3) in the gut as well as RORγt independent events (161). Simple ectopic overexpression of several chemokines is sufficient to culminate in TLT formation (152). In general, regardless of the inducing trigger, signaling by LTβR is essential for the formation of mature TLT in most settings with the exceptions being relatively small, more poorly organized structures (14, 91, 152, 162). FDC and especially HEV formation in TLT is clearly reduced following pharmacological inhibition of the LT pathway (155, 156, 162). In a mouse pancreas model, large TLT were dissociated by LT pathway inhibition, yet in the small remaining residual T cell rich zones, the FRC networks appeared fully developed with conduits (91). This result is consistent with the viewpoint that FRC maintenance is LT pathway independent.

In a major knowledge gap, the contribution of TLT to pathology is defined predominately by correlation and association. The presence of TLT in man is associated with more severe disease, e.g., in juvenile dermatomyositis, Sjogren’s syndrome, and multiple sclerosis (163–165). In rodents, TLT can enhance viral defense in lung infections, naïve T cell recruitment, and epitope spreading in diabetes and exacerbate heart allograft rejection (152, 166–168). Furthermore, HEV can be found in the absence of mature TLT and, indeed, simply the emergence of cardiac HEV is a strongly prognostic for pending heart graft rejection (169).

### Tumors

The role of the LT pathway in tumor biology has become an active area. LTβR signaling can promote tumor metastasis by maintaining a level of pro-inflammatory signaling (170–173). In preexisting tumors, the formation of HEV within tumors appears to be beneficial presumably by allowing for entry of a wider range of lymphocyte subsets and enhanced tumor immunity (174). HEV are found in melanoma tumors often in close proximity to LTβ-expressing DC suggesting that processes are in play analogous to those maintaining HEV in LN (43).

## Future Directions

A clear picture of the integration of the vascular and reticular networks in the primary, secondary, and tertiary immune organs in resting, reactive, and pathological states is essential to an understanding of how the immune system maintains optimal sensitivity and selectivity. It is also reasonable to assume that many pathological events revolve not only around altered adaptive and innate immunology, but also on altered stromal elements, e.g., HIV infection. The LT system is clearly interwoven in some of these processes, yet many of the studies have blurred the distinction between developmental and maintenance controls. More work is required especially at the clinical level to understand the contributions of the LT system in human disease and it is fortuitous that various clinical interventions are targeting LT (anti-LTα antibody), LIGHT (anti-LIGHT antibody), and both LT and LIGHT (LTβR-Ig).

Certainly studies of stromal cell states in lymphoid tissues serve to expand the basic knowledge of these differentiation programs and the reactive state is a model of controlled inflammation in a lymphocyte-rich environment. Importantly, the LN can return to the resting state – a perfect example of physiological tissue remodeling. Studying the lymphoid setting may help one comprehend the interplay between chronic inflammation, vascular damage, and stromal cell activation present in many diseases. Given the success of recent immunomodulatory strategies in oncology, manipulation of the HEV entry portals for lymphocytes as well as modification of lymphocyte–tumor stroma interactions could be very productive approaches (174, 175). There is considerable interest in the control of the differentiation pathways leading from mesenchymal lineages into the myofibroblastoid cells driving pathological tissue remodeling and fibrosis (176). For example, vascular disruption and dysfunctional pericyte-endothelial cell interaction appears to be occurring in many pathological conditions including scleroderma, interstitial lung disease, lupus, multiple sclerosis, some neurodegenerative diseases and diabetic retinopathy, to name a few (67). It is possible that important lessons can be gleaned from the analysis of these cells in lymphoid microenvironments.

## Conflict of Interest Statement

The authors declare that the research was conducted in the absence of any commercial or financial relationships that could be construed as a potential conflict of interest.
